# Prevalence of Pruritus in Cutaneous Lupus Erythematosus: Brief Report of a Multicenter, Multinational Cross-Sectional Study

**DOI:** 10.1155/2018/3491798

**Published:** 2018-07-25

**Authors:** Dominik Samotij, Justyna Szczęch, Carolyn J. Kushner, Mohammad Rafiqul Mowla, Aleksandra Dańczak-Pazdrowska, Emiliano Antiga, François Chasset, Fukumi Furukawa, Minoru Hasegawa, Hideo Hashizume, Aminul Islam, Takaharu Ikeda, Aleksandra Lesiak, Adriana Polańska, Laurent Misery, Jacek C. Szepietowski, Daisuke Tsuruta, Zygmunt Adamski, Victoria P. Werth, Adam Reich

**Affiliations:** ^1^Department of Dermatology, University of Rzeszow, Rzeszow, Poland; ^2^Perelman School of Medicine, University of Pennsylvania and Corporal Michael J. Crescenz VAMC, Philadelphia, USA; ^3^Department of Dermatology, Chittagong Medical College, Chittagong, Bangladesh; ^4^Department of Dermatology, Poznan University of Medical Sciences, Poznan, Poland; ^5^Department of Surgery and Translational Medicine, Section of Dermatology, University of Florence, Florence, Italy; ^6^Sorbonne University, Department of Dermatology and Allergology, Tenon Hospital, Paris, France; ^7^Department of Dermatology, Wakayama Medical University, Wakayama, Japan; ^8^Department of Dermatology, Division of Medicine, Faculty of Medical Sciences, University of Fukui, Yoshida-gun, Japan; ^9^Department of Dermatology, Shimada Municipal Hospital, Shimada, Japan; ^10^Department of Dermatology, Pediatric Dermatology and Oncology Clinic, Medical University of Lodz, Łódź, Poland; ^11^Department of Dermatology and Venereology, Poznan University of Medical Sciences, Poznan, Poland; ^12^Department of Dermatology, University Hospital of Brest, Brest, France; ^13^Department of Dermatology, Venereology and Allergology, Wroclaw Medical University, Wroclaw, Poland; ^14^Department of Dermatology, Osaka City University Graduate School of Medicine, Osaka, Japan

## Abstract

Pruritus is an important symptom frequently accompanying various inflammatory skin conditions. Some recent data have indicated that it may also be associated with autoimmune connective tissue diseases, including systemic sclerosis and dermatomyositis; however, studies on the prevalence and clinical characteristics of pruritus in CLE are limited. We have performed a multinational, prospective, cross-sectional study in order to assess the prevalence and intensity of pruritus in adult patients suffering from various subtypes of CLE. After developing a questionnaire assessing various aspects of pruritus, we have surveyed 567 patients with cutaneous involvement during the course of LE regarding the presence and intensity of pruritus. Pruritus was present in 425 of all patients (75.0%) and was most frequently reported by subjects with acute CLE (82.1%), followed by chronic CLE (78.8%), subacute CLE (65.9%), and intermittent CLE (55.6%) (p<0.001). Based on the Numerical Rating Scale, the severity of itch was mild, moderate, and severe in 264 (62.1%), 98 (23.1%), and 63 (14.8%) patients, respectively. The highest mean pruritus intensity was reported by subjects with hypertrophic LE (5.1 ± 3.0 points) followed by generalized discoid LE (3.6 ± 3.0 points), subacute CLE (3.0 ± 3.0 points), chilblain LE (3.0 ± 1.0 points), localized discoid LE (2.6 ± 2.0 points), intermittent CLE (2.6 ± 3.0 points), acute CLE (2.5 ± 1.2 points), and lupus erythematosus profundus (1.9 ± 2.7 points). In conclusion, pruritus is a frequent phenomenon in CLE; however, in most patients it is of mild severity. Further studies are needed to better characterize its clinical characteristics and influence on patients' well-being.

## 1. Introduction

Cutaneous lupus erythematosus (CLE) is an autoimmune condition encompassing a wide range of dermatologic manifestations. Skin involvement in CLE patients can be divided into two categories based on histology: lupus erythematosus- (LE-) specific and LE-nonspecific skin lesions. The presence of LE-specific lesions is necessary to confirm the diagnosis of CLE. LE-specific skin lesions are divided into several subtypes based on clinical characteristics: acute CLE (ACLE), subacute CLE (SCLE), and chronic CLE (CCLE) with several variants including discoid LE (DLE), presenting as a localized or generalized form, LE profundus (LEP) (also called lupus panniculitis or subcutaneous LE), hypertrophic LE (HLE), chilblain LE (CHLE), and lupus erythematosus tumidus (LET) [1]. Some authors consider LET as a distinct subtype, namely, intermittent CLE (ICLE) [2]; thus, this subtype was considered separately in our analysis.

Pruritus is defined as an unpleasant sensation causing a desire to scratch [3]. Pruritus is regarded as the most prevalent subjective symptom in dermatology reported by more than half of the patients with skin disorders [4]. Its high prevalence in certain dermatological conditions, such as atopic dermatitis or urticaria, has been widely confirmed and thoroughly investigated in terms of quantitative and qualitative measures [5, 6]. In some other very common skin disorders like psoriasis, it was only recent years that confirmed its actual importance in terms of high prevalence and marked impact on patients' quality of life (QoL) [7]. A recent data suggests that itch may also be a common component of autoimmune connective tissue diseases, including systemic sclerosis (SSc) and dermatomyositis (DM) [8–10]. However, studies on the true prevalence and clinical characteristics of pruritus in CLE are very limited and bring ambiguous results. Discrepancies between various studies that included itch evaluation in LE patients with cutaneous involvement may suggest that, without adequate investigation by medical personnel, the presence of this bothersome symptom could easily be underestimated or even missed. Therefore, we performed a multinational, multicenter study to precisely assess the prevalence and clinical characteristics of pruritus in different variants of CLE in relation to skin lesion spectrum.

## 2. Methods

A multinational, multicenter, prospective, cross-sectional study was conducted in order to determine the prevalence of pruritus and assess its intensity and clinical characteristics in adult patients suffering from various subtypes of CLE. Centers from various continents with special interest and experience in CLE diagnostics and treatment were selected to cover possible racial and environmental differences: 6 centers from 3 countries in Europe (France, Italy, Poland), 5 centers from 2 countries in Asia (Japan, Bangladesh), and 1 center from North America (Pennsylvania). The study was conducted in accordance with the Data Protection Act and according to the ethical guidelines of the Declaration of Helsinki and was approved by the Ethics Committee at Wroclaw Medical University in Poland.

### 2.1. Patients 

A total number of 567 inpatients and outpatients of aforementioned centers (Asia: n=119, 21.0%, Europe: n=55, 9.7%, and North America: n=393, 69.3%) with cutaneous involvement during the course of LE were included in the study. Only patients with active skin lesions were included. Diagnosis of CLE has been established based on clinical manifestation and skin biopsy, if necessary. Only patients with CLE specific lesions were included into this study according to Sontheimer [1] and Kuhn et al. [2]. Patients with other skin diseases, which might influence the achieved results, were excluded.

All patients agreed to participate in the study. Their age ranged between 18 and 89 years (mean 41.4 ± 13.2 years), and 472 patients (83.2%) were women. Among analyzed subjects, 330 (58.2%) patients were diagnosed as having CCLE (including 302 patients with DLE, 14 patients with LEP, 11 patients with HLE, and 3 patients with CHLE), 123 (21.7%) as having SCLE, 78 (13.8%) as having ACLE, and 36 (6.3%) with ICLE (LET). The mean disease duration was 9.8 ± 11.2 years. The disease activity and damage of CLE were assessed according to the Cutaneous Lupus Erythematosus Disease Area and Severity Index (CLASI) [11].

### 2.2. Study Instruments and Assessments

The current report describes results on the evaluation of prevalence and severity of pruritus in CLE patients being a prerequisite for further investigation focusing on the clinical manifestation of pruritus in CLE patients and its influence on the patients' quality of life. After receiving a formal approval to participate and collecting basic demographic data, all patients with CLE were asked whether they suffer from pruritus or not. Only patients with pruritus lasting ≥6 weeks were considered as positive. If the answer was “yes”, the patient was asked to assess the average severity of pruritus within the previous 3 days using the Numerical Rating Scale from 0 to 10. All patients were instructed that 0 means no itch while 10 means the worst imaginable itch. Subsequently, pruritus intensity was categorized with the following cut-off values: 0 points: no pruritus, 1-3 points: mild pruritus, 4-6 points: moderate pruritus, and 7-10 points: severe pruritus.

### 2.3. Statistical Analysis

All results were analyzed statistically using Statistica® 12.0 (Statsoft, Krakow, Poland). Descriptive statistics included frequencies, mean, standard deviation, median, minimal, and maximal values. The significance of the observed differences between groups has been determined by Mann–Whitney U test or *χ*^2^ test with Yates correction, where necessary. Correlation between studied parameters were verified with Spearman rank correlation test. A *p* value lower than 0.05 was considered as statistically significant.

## 3. Results

### 3.1. Prevalence of Pruritus in CLE Subtypes

Pruritus was present in 425 of all analyzed patients (75.0%). It was most frequently reported by subjects with ACLE (82.1%), followed by CCLE (78.8%), SCLE (65.9%), and ICLE (55.6%) (p<0.001). According to the International Forum for the Study of Itch classification [3], pruritus in CLE subjects was considered as dermatological one. In 85.1% of patients pruritus was limited to the skin lesions, while in 14.9% also uninvolved skin was itchy.

### 3.2. Severity of Pruritus in CLE Subtypes

**Figure 1:**
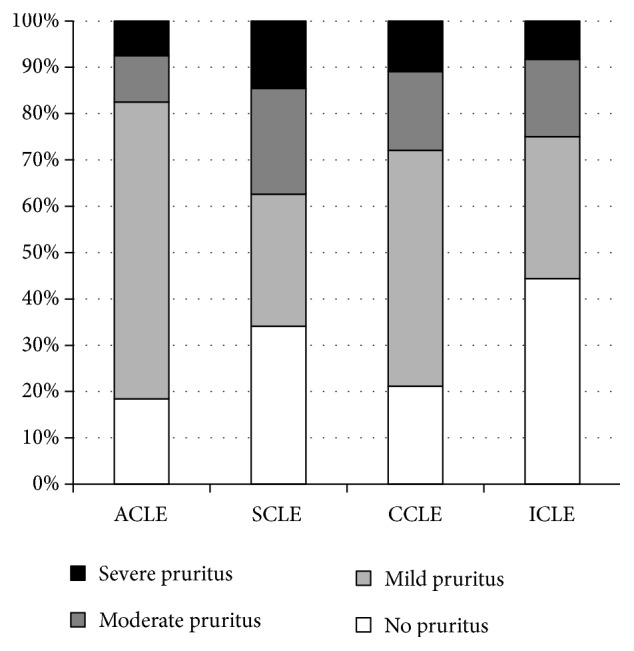
Prevalence of pruritus in various subtypes of cutaneous lupus erythematosus (p=0.002).

Based on the NRS, the severity of itch was mild, moderate, and severe in 264 (62.1%), 98 (23.1%), and 63 (14.8%) patients reporting this symptom, respectively. With regard to the specific subtypes of CLE, in ACLE mild pruritus was found in 50 (64.1%) patients, moderate in 8 (10%), and severe in 6 (7.5%), and in SCLE mild pruritus was reported by 35 (28.5%) patients, moderate by 28 (22.8%), and severe by 18 (14.6%), and in CCLE mild pruritus was found in 168 (50.9%) patients, moderate in 56 (17%), and severe in 36 (10.9%), while among ICLE subjects mild pruritus was reported by 11 (30.6%) patients, moderate by 6 (16.7%), and severe by 3 (8.3%), respectively (p=0.002) (Figure 1). A significant correlation was observed between pruritus intensity and activity of skin lesions according to CLASI (*ρ*=0.45, p<0.001), but not with the skin damage scoring (*ρ*=-0.07, p=0.51). The age and CLE duration did not influence significantly the pruritus intensity reported by the patients (*ρ*=-0.06, p=0.58 and *ρ*=-0.16, p=0.13, respectively).

### 3.3. Severity of Pruritus in CCLE Variants

The severity of itch among subjects with localized DLE was mild in 126 (61.5%) patients, moderate in 26 (12.2%), and severe in 14 (6.8%). Among subjects with generalized DLE, the severity of itch was mild in 29 (29.9%) patients, moderate in 26 (26.8%), and severe in 18 (18.6%). Among subjects with LEP, the severity of itch was mild in 5 (35.7%) patients, moderate in 2 (14.3%) patients, and severe in 1 (7.1%) patient, while in HLE the severity of itch was mild in 5 (45.4%) patients and moderate and severe in 3 (27.3% each) (Table 1). All 3 subjects with CHLE reported mild pruritus (p<0.001).

### 3.4. Mean Pruritus Intensity

The highest mean NRS value was reported by subjects with HLE (5.1 ± 3.0 points) followed by generalized DLE (3.6 ± 3.0 points), SCLE (3.0 ± 3.0 points), CHLE (3.0 ± 1.0 points), localized DLE (2.6 ± 2.0 points), ICLE (2.6 ± 3.0 points), ACLE (2.5 ± 1.2 points), and LEP (1.9 ± 2.7 points) patients.

## 4. Discussion

Our data suggests that pruritus is indeed a common subjective symptom of CLE, as it was present on average in 3 out of 4 patients included in our study, albeit, in general, it was of mild severity. The prevalence of severe pruritus was about 10% depending on CLE subtypes. Similarly, Kapadia and Haroon [8] studied Indian patient population with systemic LE (SLE) and confirmed that itch was a frequent feature of the disease with a reported prevalence of 45%. However, the study had a major limitation since it only included patients with SLE rather than those with CLE. In addition, the groups were not diversified based on LE-specific and LE-nonspecific lesions, making it impossible to establish the possible relation of pruritus to the presence, pattern, and intensity of cutaneous involvement [8]. On the other hand, Goreshi et al. [9] investigated QoL in DM in comparison to CLE and found the severity of itch to be higher in DM than in CLE. They suggested that in certain cases the presence of pruritus should exclude the diagnosis of CLE rather than confirm it. This conclusion suggested that itch is not a major complaint in CLE. This study did not, however, provide information about the prevalence of pruritus in these subsets of patients [9]. An interesting, yet rather small observational study was performed in CLE subjects treated at the Hospital of the University of Pennsylvania, Philadelphia. The aim was to measure the correlation of change in Cutaneous Lupus Erythematosus Disease Area and Severity Index (CLASI™) with the change in patients' assessments of pain and pruritus 14 weeks after starting a new standard of care therapy. However, the correlation of CLASI score with the patient's assessment of itch level was only moderate. Not every patient complained of itch, and some patients actually developed this symptom independently of the skin condition and the treatment. Therefore, the authors concluded that as it did not reflect disease progression or severity, it was unlikely that pruritus could be a part of reliable disease activity measurement in CLE [11]. A more recent study performed by Méndez-Flores et al. [12] found no difference in pruritus score between patients who had CLE, LE-nonspecific skin manifestations, or a combination of the two. However, the authors found a median pruritus VAS score of 6 points in the LE-specific disease group, 4 points in the LE-nonspecific disease group, and 6.5 points in the combination group. Although the differences in scores between groups were not significant, these data do suggest that patients with LE-specific skin disease in particular do experience a moderate level of pruritus. Additionally, in the group with LE-specific lesions, the pruritus score and the CLASI activity score were correlated (p=0.01), suggesting that itch is a symptom of active CLE lesions. However, in this study, pain but not itch intensity was significantly correlated with QoL [12].

Comparing our observations with the available data on the frequency of itching in other connective tissue diseases, we can state that this symptom is substantially more prevalent in CLE than in SLE (45%) and SSc (43%). Much to our surprise, its frequency was even comparable to pruritus prevalence observed in other studies on psoriasis (70-90% of patients), late stage cutaneous T cell lymphoma (83%) [13], or chronic idiopathic urticaria (79%) [6]. Considering a small number of systematic studies on CLE that include pruritus as one of the evaluated domains, and a lack of research dedicated exclusively to the assessment of itch qualities in these patients, it seems to be a frequently overlooked and/or underappreciated symptom of this condition. Based on our results we may state that the highest prevalence rate of pruritus was observed in subjects with ACLE. Assumption that in the most inflammatory skin phenotype the presence of itch is a reflection of more active cutaneous lesions would be the most obvious explanation of the highest itch prevalence in subjects suffering from this particular CLE subtype. Surprisingly, it stands in contradiction to the other published results that show a relatively low prevalence of pruritus in SLE patients [8, 14]. Nonetheless, CCLE sufferers reported pruritus surprisingly often, which was also contradictory to our expectations. However, numerous case reports and review articles on this CLE variant mention pruritus, along with pain, as accompanying symptom of the disease. One of the hypotheses that would explain the high prevalence of these symptoms in this particular CLE subtype is the compression of cutaneous nerves secondary to dermal fibrosis, in a manner somewhat similar to that observed in late stage SSc. ICLE patients complained of itching significantly less often than CCLE subjects (55,6% versus 78,8%). This observation, among other distinct features of ICLE and its differences from CCLE, may serve as another point for including ICLE as a separate (fourth) subtype of CLE, as proposed by A. Kuhn et al. [2]. Of note, more recent studies have shown a pathogenic role of interleukin 18 (IL-18) [15] in CLE and significance of toll-like receptor 7 (TLR7) [16] in SLE. Both proteins are known to play a role in the induction of pruritus [17, 18].

Several limitations should be considered when interpreting the results of this study. The major one is a lack of longitudinal prospective design and that the patients were assessed in different stages of treatment which could have influenced their responses regarding pruritus. Some medications can affect the intensity of itching that is experienced by patients. Another contribution to the possible bias of the results could be the fact that most of the patients were treated in highly specialized academic centers. It is thus possible that these patients seek treatment or were referred due to earlier therapeutic difficulties, possibly with pruritus as more prominent symptom than in less selective patient population. However, our study required participation of physicians qualified and experienced in treating CLE patients who are usually linked to academic centers. Finally, lack of a control group is another limitation which precluded us to compare the prevalence of pruritus in CLE patients with reference to the general population.

## Figures and Tables

**Table 1:** Intensity of pruritus in various subtypes of chronic cutaneous lupus erythematosus.

	No pruritus	Mild pruritus	Moderate pruritus	Severe pruritus
Localized discoid lupus erythematosus	19,5%	61.5%	12.2%	6.8%

Generalized discoid lupus erythematosus	24,7%	29.9%	26.8%	18.6%

Lupus erythematosus profundus	42,9%	35.7%	14.3%	7.1%

Hypertrophic lupus erythematosus	0%	45.4%	27.3%	27.3%

Chilblain lupus erythematosus	0%	100%	0%	0%

## Data Availability

The data used to support the findings of this study are available from the corresponding author upon request.
